# Pi-pi Stacking Mediated Cooperative Mechanism for Human Cytochrome P450 3A4

**DOI:** 10.3390/molecules20057558

**Published:** 2015-04-24

**Authors:** Botao Fa, Shan Cong, Jingfang Wang

**Affiliations:** 1 Key Laboratory of Systems Biomedicine (Ministry of Education), Shanghai Center for Systems Biomedicine, Shanghai Jiao Tong University, Shanghai 200240, China; E-Mail: faber@sjtu.edu.cn; 2 Department of Bioinformatics and Biostatistics, College of Life Sciences and Biotechnology, Shanghai Jiao Tong University, Shanghai 200240, China; E-Mail: congshan8113@sjtu.edu.cn

**Keywords:** Cytochrome P450, drug metabolism, cooperative binding, pi-pi stacking

## Abstract

Human Cytochrome P450 3A4 (CYP3A4) is an important member of the cytochrome P450 superfamily with responsibility for metabolizing ~50% of clinical drugs. Experimental evidence showed that CYP3A4 can adopt multiple substrates in its active site to form a cooperative binding model, accelerating substrate metabolism efficiency. In the current study, we constructed both normal and cooperative binding models of human CYP3A4 with antifungal drug ketoconazoles (KLN). Molecular dynamics simulation and free energy calculation were then carried out to study the cooperative binding mechanism. Our simulation showed that the second KLN in the cooperative binding model had a positive impact on the first one binding in the active site by two significant pi-pi stacking interactions. The first one was formed by Phe215, functioning to position the first KLN in a favorable orientation in the active site for further metabolism reactions. The second one was contributed by Phe304. This pi-pi stacking was enhanced in the cooperative binding model by the parallel conformation between the aromatic rings in Phe304 and the dioxolan moiety of the first KLN. These findings can provide an atomic insight into the cooperative binding in CYP3A4, revealing a novel pi-pi stacking mechanism for drug-drug interactions.

## 1. Introduction

Cytochrome P450s (CYPs) belongs to a large group of heme-containing mono-oxygenases [1]. Most of these enzymes contain a heme group in the protein center, which employed therein absorbs light at the 450 nm wavelength after being complexed with carbon monoxide. Thus, these enzymes are so-named “P450”. Cytochrome P450s can be detected in all kinds of organisms, acting as metabolizing enzymes for both endogenous and exogenous compounds [2]. As reported, Mammalian CYP enzymes are major membrane-associated proteins located in either the endoplasmic reticulum or the inner membrane of the mitochondria of cells [3]. Due to the ability of metabolizing both xenobiotics and endogenous compounds, these enzymes are considered essential for the detoxification of exogenous substances and the control of endogenous compound levels. In human, CYP proteins are the major drug-metabolizing enzymes in liver cells with responsibility for phase I drug metabolism [4]. Over 90% of marketed and clinical drugs are metabolized by CYP enzymes [4].

Human cytochrome P450 3A4 (CYP3A4) is an important member of cytochrome P450 superfamily. It is the most abundant CYP in human liver, and is also the most common and most versatile CYP enzyme involved in drug metabolism. CYP3A4 is predominantly expressed in the liver cells and at low levels in intestine, gut, colon, prostate, and brain [5]. In human cells, CYP3A4 protein is located on the endoplasmic reticulum, functioning as a major metabolizing enzyme for approximately 50% of marketed medicines, as well as some steroids and carcinogens [6]. Additionally, this CYP enzyme is significantly involved in the metabolism of the immunosuppressive cyclic peptide cyclosporine A and macrolide antibiotics. CYP3A4 can perform an assortment of modifications on a wide range of substrates by hydroxylation, aromatic oxidation, heteroatom oxidation, and dealkylation reactions [7]. Like other CYP enzymes, CYP3A4 has many individual variations, playing crucial roles in breast and prostate carcinogenesis through modulation of sex hormone and metabolite level [8].

Usually, enzymes can adopt only one substrate in their active site. However, more and more evidence shows that the substrate-binding mode of CYP3A4 does not fit Michaelis–Menten type kinetics, showing atypical steady-state kinetics *in vitro* and presumably *in vivo* [9]. This observation gives an indication that CYP3A4 can bind two or more substrates in the active site simultaneously (so-called cooperative binding) [9,10,11,12]. By now, many theoretical and computational studies have been performed on the cooperative binding mode of CYP3A4 [13,14,15,16]. According to Fishelovitch and coworkers [15], due to the large active site volume, both substrates can simultaneously bind in the active pocket. The second substrate has direct interactions with the first one, stabilizing the binding mode of the first substrate in the active site and enhancing a favorable orientation of the first substrate for oxidation.

Recently, we reported a novel mechanism for the cooperative binding of human CYP2E1 [17,18]. This CYP enzyme can also adopt two substrates. However, due to the small active site volume, only one substrate can bind in the active site, with another substrate in the second binding site [19]. The second substrate can influence the metabolism of the first one via pi-pi stacking interactions. As both CYP3A4 and CYP2E1 have cooperative binding properties, what role does pi-pi stacking play in the cooperative binding of CYP3A4? To answer this, we constructed both normal and cooperative binding models for CYP3A4 based on the crystal structures 1tqn and 1w0f (so-named CYP3A4t and CYP3A4w structures). Molecular dynamics simulation and free energy calculation were applied to analyze the dynamic behaviors of these binding modes and the free energy contributions of each residue in the active site.

## 2. Results and Discussion

### 2.1. General Analysis of Molecular Dynamics Trajectories

Crystal structures 1tqn and 1w0f are the most applied structures for human CYP3A4 to study the dynamics behaviors of cytochrome P450s [7,20,21]. However, the two structures are not the identical. The main difference between 1tqn and 1w0f is the orientation of Arg212: one is toward the heme group (1tqn) and the other is facing out of the active site (1w0f). The different orientation of Arg212 will cause distinct side chain interactions, which have been thought to play crucial roles in substrate binding and metabolism. In our simulations, a strong interaction between Arg212 and Phe304 was detected in the CYP3A4t model, making Arg212 face toward the active site. However, in the CYP3A4w model, this residue formed a salt bridge with Glu308, thus making it face out of the active site. The different side chain orientations lead to different KLN binding modes, so we involved both crystal structures in constructing the normal and cooperative binding models.

**Figure 1:**
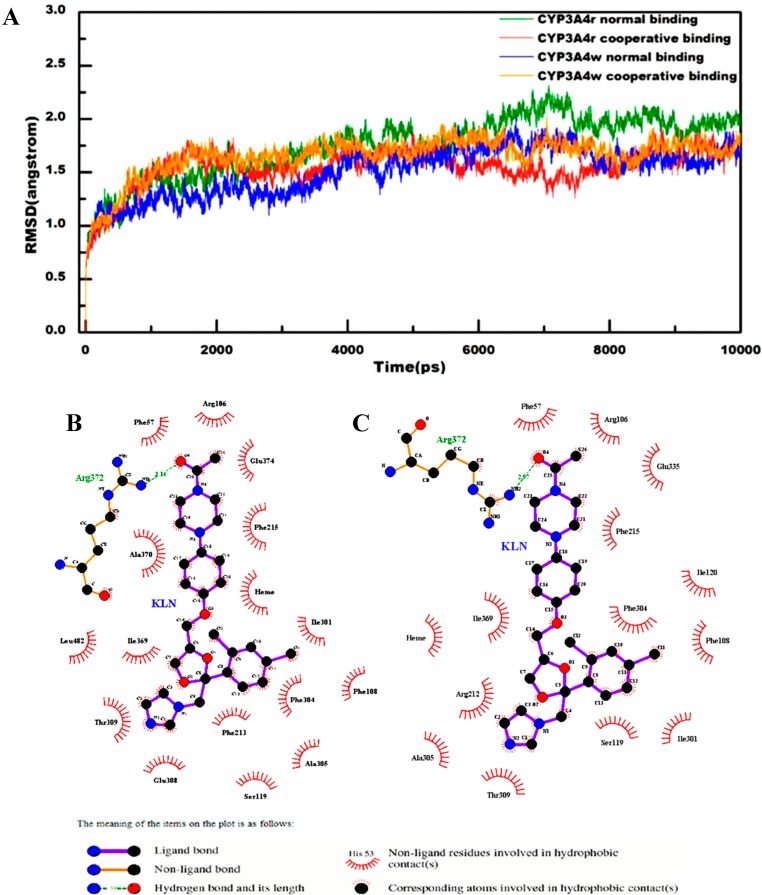
(**A**) RMS deviations for protein backbone structure of both normal and cooperative binding models in CYP3A4t and CYP3A4w structures; (**B**) 2D-diagram of the detailed binding information for the normal binding model in CYP3A4t structure; and (**C**) 2D-diagram of the detailed binding information for the normal binding model in CYP3A4w structure.

All the computational models involved in the current study were summarized in Table S1. To measure the convergence of these computational models in molecular dynamics simulations, we calculated the root-mean-square (rms) deviations of the protein backbone structures for all computational models. As shown in Figure 1A, the final rms deviations for all the computational models were no more than 2.5 Å. As expected, no significant fluctuations for rms deviations were detected for all the computational models. These observations indicated that the computational models in the current study were all equilibrated. Interestingly, the rms deviations for the normal binding models in CYP3A4t and CYP3A4w structures had large difference. But the rms deviations for the cooperative binding models in CYP3A4t and CYP3A4w structures were quite similar, suggesting that Arg212 might adopt similar conformation in the cooperative binding for both CYP3A4t and CYP3A4w structures.

Indeed, there is another crystal structure of human CYP3A4 (PDB entry 2v0m), which employs two ketoconazole molecules binding in the active site to form a cooperative binding model. For comparison, we calculated the rms deviations of the protein backbone structures for CYP3A4t and CYP3A4w structures to the crystal structure 2v0m. For the CYP3A4t structure, the rms deviations to the crystal structure 2v0m were 1.09 ± 0.94 Å and 1.03 ± 0.89 Å for the normal and cooperative binding models, while those for the CYP3A4w structure were 6.80 ± 1.10 Å (normal binding model) and 6.55 ± 1.03 Å (cooperative binding model). This finding indicated that the crystal structure 2v0m was quite close to the CYP3A4t structure, but different from the CYP3A4w structure.

### 2.2. Interactions for Normal and Cooperative Binding

In the normal binding model, Arg372 formed a strong hydrogen bond with KLN O4 atom in both CYP3A4t and CYP3A4w structures (Figure 1B,C). This hydrogen bonding interaction, together with the hydrophobic interaction contributed by Phe215 and piperazine moiety of KLN, functioned as forceps to recognize and position substrates in a favorable binding site and orientation for further metabolism reactions. The interactions near KLN dioxolan moiety in CYP3A4t and CYP3A4w structures were quite different. In the CYP3A4t structure, the imidazole group in KLN dioxolan moiety was stabilized by Glu308 and Thr309 (Figure 1B). However, in the CYP3A4w structure, Ala305 and Arg212 together with Thr309 formed hydrophobic interactions with the imidazole group of KLN dioxolan moiety (Figure 1C). For the phenyl group of KLN dioxolan moiety, Phe108, Ile301 and Phe304 were found to have strong hydrophobic interactions in both CYP3A4t and CYP3A4w structures. Besides, more residues, *i.e.*, Ala305, Ser119 and Phe213 were detected to be involved in the hydrophobic interactions in the CYP3A4t structure. Additionally, the average distances between the imidazole nitrogen of KLN and the iron in the heme group were 5.24 Å for the CYP3A4t structure and 5.60 Å for the CYP3A4w structure.

**Figure 2:**
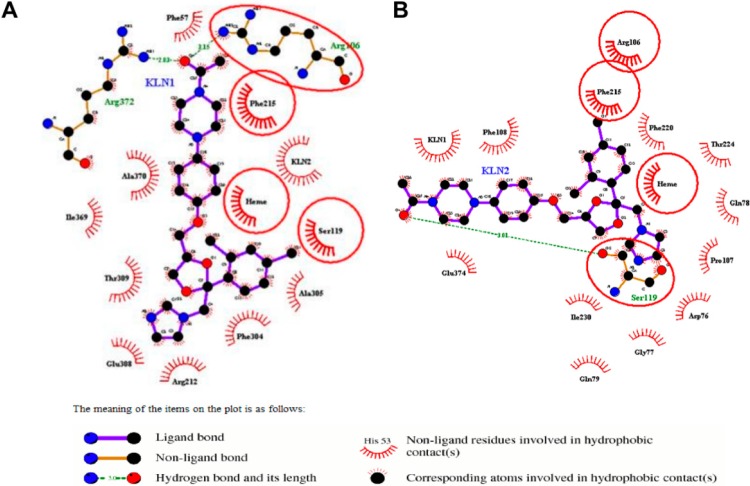
2D-diagram of the detailed binding information for the (**A**) normal and (**B**) cooperative binding model of CYP3A4t structure. The first KLN binding in the active site was labeled as KLN1, and the second one as KLN2.

**Figure 3:**
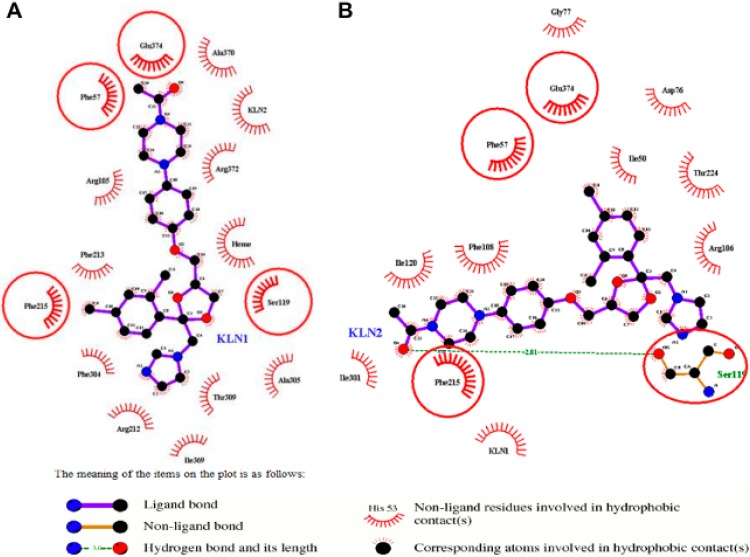
2D-diagram of the detailed binding information for the (**A**) normal and (**B**) cooperative binding model of CYP3A4w structure. The first KLN binding in the active site was labeled as KLN1, and the second one as KLN2.

In the cooperative binding of the CYP3A4t structure, forceps, used for recognizing and positioning substrates, as mentioned above, were strengthened by more hydrogen bonding and hydrophobic interactions. Besides Arg372, Arg106 was also found to have hydrogen-bonding interactions with KLN O4 atom (Figure 2A). Phe57 and Phe215 provided significant hydrophobic interactions. Additionally, the hydrogen bond between Arg372 and KLN O4 atom in the CYP3A4t structure became stronger, as its bond length (~2.83 Å) was a little shorter than the normal binding model (~3.16 Å). In the cooperative binding of the CYP3A4w structure, the hydrogen bond formed by Arg372 and the hydrophobic interaction formed by Phe215 disappeared. Instead, Phe57, Ala370, Arg372, Glu374 as well as the second KLN formed a new hydrophobic interaction network to stabilize the piperazine moiety of the first KLN (Figure 3A). The average distance between the imidazole nitrogen of the first KLN and the iron in the heme group were a little smaller in the cooperative binding models than those in the normal binding models. In the cooperative binding model, the aforementioned average distance was 3.62 Å for the CYP3A4t structure and 3.86 Å for the CYP3A4w structure, respectively.

For the second KLN molecule, Ser119 formed a hydrogen bond with the O4 atom in the second KLN piperazine moiety in both CYP3A4t and CYP3A4w structures (Figure 2B and Figure 3B). This hydrogen bond, together with Glu374 and the first KLN in the CYP3A4t structure (Ile120, Phe215 and Ile301 in the CYP3A4w structure), played an important role in recognizing the second substrate for human CYP3A4. Similar with the first KLN molecule, the dioxolan moiety of the second KLN was stabilized by multiple hydrophobic interactions. In the CYP3A4t structure, the aforementioned hydrophobic interactions were formed by residues 76–79, Arg106, Phe107, Phe215, Phe220 and Thr224 (Figure 2B). While in the CYP3A4w structure, Phe57, Arg106, Thr224 and Glu374, were mainly involved in the hydrophobic interactions with the dioxolan moiety of the second KLN (Figure 3B). All the residues mentioned above made up the second binding pocket for the second substrate in human CYP3A4.

### 2.3. Binding Free Energy Analysis

To get an in-depth understanding of the CYP3A4-KLN interactions, MM-PB/SA and MM-GB/SA approaches were employed to estimate the binding free energies for both normal and cooperative binding models. For the CYP3A4t structure, the binding free energies for the first KLN molecule in the normal binding model were −38.86 ± 4.00 kcal/mol (MM-PB/SA) and −54.41 ± 2.64 kcal/mol (MM-GB/SA), while those for the cooperative binding model were −47.56 ± 4.36 kcal/mol (MM-PB/SA) and −66.95 ± 3.12 kcal/mol (MM-GB/SA), respectively (Table 1). For the CYP3A4w structure, the binding free energies for the first KLN molecule in the normal binding model were −29.83 ± 4.26 kcal/mol (MM-PB/SA) and −50.97 ± 3.22 kcal/mol (MM-GB/SA), while those for the cooperative binding model were −44.50 ± 4.45 kcal/mol (MM-PB/SA) and −66.81 ± 3.74 kcal/mol (MM-GB/SA), respectively (Table 1).

The binding free energy differences between normal and cooperative binding models were −15.55 kcal/mol in the CYP3A4t structure and −19.39 kcal/mol in the CYP3A4w structure estimated by MM-PB/SA approach, while the difference for MM-GB/SA approach were −21.14 kcal/mol in the CYP3A4t structure and −22.31 kcal/mol in the CYP3A4w structure. These differences were significant in statistics, as their p values for fisher test were all less than 0.05. This observation indicated that the second KLN molecule had a positive impact on the first KLN binding in the active site.

**Table 1:** Binding free energy for the first KLN binding in the active site based on MM-PB/SA and MM-GB/SA methods for the normal and cooperative binding models of human CYP3A4.

Energies (kcal/mol)	CYP3A4t		CYP3A4w	
	Normal	Cooperative	Normal	Cooperative
Δ*E*_ele_	−20.80 ± 2.71	−25.68 ± 3.08	−31.63 ± 3.45	−25.29 ± 3.57
Δ*E*_vdw_	−65.33 ± 2.46	−74.99 ± 2.57	−57.44 ± 2.37	−75.63 ± 3.42
Δ*E*_gas_	−86.13 ± 3.34	−100.67 ± 3.68	−89.06 ± 3.17	−100.92 ± 4.35
Δ*G*_nonpolar/PB_	−8.37 ± 0.21	−8.44 ± 0.14	−7.79 ± 0.06	−7.84 ± 0.15
Δ*G*_sol/PB_	47.27 ± 3.84	53.11 ± 3.15	59.24 ± 2.46	56.43 ± 2.67
Δ*G*_ele/PB_	34.84 ± 3.64	35.87 ± 4.17	35.40 ± 4.15	38.97 ± 3.86
Δ*G*_bind/PB_	−38.86 ± 4.00	−47.56 ± 4.36	−29.83 ± 4.26	−44.50 ± 4.45
Δ*G*_nonpolar/GB_	−8.37 ± 0.21	−8.44 ± 0.14	−7.79 ± 0.16	−7.84 ± 0.15
Δ*G*_sol/GB_	31.72 ± 2.50	33.72 ± 2.21	38.10 ± 2.85	34.11 ± 2.04
Δ*G*_ele/GB_	19.29 ± 2.23	16.48 ± 2.44	14.26 ± 2.44	16.65 ± 2.82
∆ *G*_bind/GB_	−54.41 ± 2.64	−66.95 ± 3.12	−50.97 ± 3.22	−66.81 ± 3.74

According to the energy decomposition in Table 1, the variations of the binding free energies for normal and cooperative binding models were mainly focused on the electrostatic energy (∆Eele) and van der Waals interactions (∆Evdw). For the CYP3A4t structure, the free energy variations were −4.88 kcal/mol and −9.66 kcal/mol for the electrostatic energy and van der Waals interactions for normal and cooperative binding models, respectively. Contrary to this, the free energy difference on the electrostatic energy for normal and cooperative binding models was 6.34 kcal/mol in the CYP3A4w structure. But the free energy difference on van der Waals interaction in this model was −18.19 kcal/mol.

### 2.4. Close Contact Analysis

**Figure 4:**
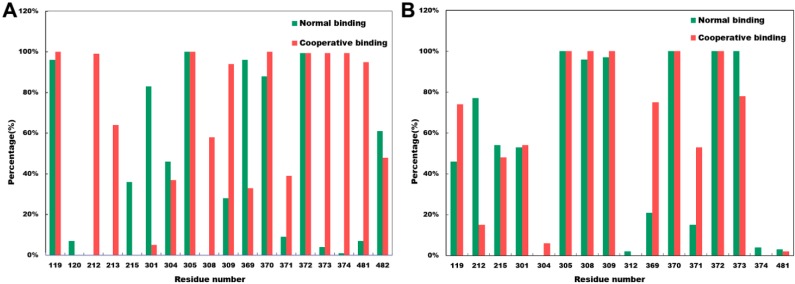
Percentage of frames over the simulation trajectories that have close contacts of less than 5 Å between the first KLN and the residues in the active site for both normal and cooperative binding models in (**A**) CYP3A4t structure and (**B**) CYP3A4w structure.

The significant free energy differences on the van der Waals interaction in both CYP3A4t and CYP3A4w structures were mainly caused by the different residue surrounding near the substrate. To characterize the residue surroundings near the first KLN in the active site, we defined that residues less than 5 Å from the first KLN had significantly close contacts with the first KLN. For both CYP3A4t and CYP3A4w structures, the significantly close contacts were found in residues 119–215, 301–309, 369–374, as well as 481–482 (Figure 4). These residues were mainly located on the regions that had structural fluctuations during our molecular dynamics simulations (Figure S1). For comparison, we also calculated the root-mean-square (rms) fluctuations of the apo form of crystal structure 2v0m, as shown in Figure S1. Different from some other enzymes, *i.e.*, pyranose dehydrogenase (PDH), the rms fluctuations of both normal and cooperative binding models were lower than the apo forms. However, according to previous work, the rms fluctuations of PDH obtained from molecular dynamics simulations were in good agreement with the crystal structure B factors [22].

For the CYP3A4t structure, the close contacts formed by Arg212, Phe213, Gly306 and Glu374 were only detected in the cooperative binding model (Figure 4A). In the cooperative binding model, the side chain orientation of Arg212 was quite different from the normal binding model: Arg212 faced to the imidazole group of the dioxolan moiety of the first KLN, forming strong hydrophobic interactions (Figure 2A). As near to Arg212, Phe213 played an important role to stabilize Arg212 in the imidazole-facing conformation.

The residues near Gly306, *i.e.*, Phe304 and Ala305, formed multiple hydrophobic interactions to stabilize the phenyl group of the dioxolan moiety of the first KLN in the cooperative binding model. Thus, the close contact formed by Gly306 might have significant impacts on these hydrophobic interactions. To support this point, we calculated the residue contributions for substrate binding free energies. As shown in Table 2, the residue contributions for Phe304 and Ala305 were −2.14 kcal/mol and −1.30 kcal/mol in the cooperative binding model, having a substantial increase than the normal binding model (−1.56 kcal/mol and −0.94 kcal/mol).

The close contact form by Glu374 was considered to be crucial in the cooperative binding model. This residue was located near Arg372, which had a significant hydrogen bond with the O4 atom of the first KLN molecule. The close contact contributed by Glu374 could enhance the aforementioned hydrogen bond by reducing the bond length to 2.83 Å (in the normal binding model, this hydrogen bond length was 3.16 Å as shown in Figure 1B). Furthermore, this close contact could also increase the binding free energy contribution for Glu374. As shown in Table 2, the residue contribution of Glu374 for the substrate binding free energy in the cooperative binding model was −1.35 kcal/mol, almost 1.24 fold of the normal binding model (−1.09 kcal/mol).

For CYP3A4w structure, Ser119, Phe304, Ile369 and Met371 employed more close contacts in the cooperative binding model (Figure 4B). The more close contact of Ser119 could enhance the hydrophobic interactions between this residue and the phenyl group of the dioxolan moiety of the first KLN molecule, leading to a greater residue contribution on the substrate binding free energy (−0.78 kcal/mol in the cooperative binding model, 1.86-fold of the normal binding model, as shown Table 3). However, the close contacts of Phe304, Ile369 and Met371 went against the substrate binding. The residue contribution for Phe304, Ile369 and Met371 on the substrate binding free energy significantly decreased in the cooperative binding model (Table 3).

**Table 2:** Binding free energy decomposition for each residue located in the active pocket of the normal and cooperative binding models constructed based on the crystal structure 1tqn.pdb.

Res. No.	Res. Name	ΔG Contribution		SRS Reg.
		Normal	Cooperative	
57	Phenylalanine	−0.79 ± 0.38	−0.96 ± 0.33	1
106	Arginine	−0.89 ± 0.33	−2.79 ± 0.76	1
108	Phenylalanine	−0.86 ± 0.27	0.04 ± 0.05	1
212	Arginine	−1.75 ± 0.37	−1.71 ± 0.29	2 and 3
215	Phenylalanine	−2.03 ± 0.35	−1.76 ± 0.30	2 and 3
301	Isoleucine	−0.80 ± 0.20	−0.71 ± 0.18	4
304	Phenylalanine	−1.56 ± 0.34	−2.14 ± 0.31	4
305	Alanine	−0.94 ± 0.25	−1.30 ± 0.26	4
308	Glutamic acid	−0.92 ± 0.37	−0.62 ± 0.34	4
309	Threonine	−1.34 ± 0.29	−1.19 ± 0.47	4
369	Isoleucine	−1.39 ± 0.32	−1.63 ± 0.33	5
370	Alanine	−1.59 ± 0.37	−1.04 ± 0.41	5
371	Methionine	−0.26 ± 0.07	−1.24 ± 0.36	5
372	Arginine	−4.31 ± 0.69	−4.25 ± 0.79	5
373	Leucine	−0.83 ± 0.17	−0.38 ± 0.11	5
374	Glutamic acid	−1.09 ± 0.70	−1.35 ± 0.45	5
482	Leucine	−1.03 ± 0.22	−0.60 ± 0.15	6

**Table 3:** Binding free energy decomposition for each residue located in the active pocket of the normal and cooperative binding models constructed based on the crystal structure 1w0f.pdb.

Res. No.	Res. Name	ΔG Contribution		SRS Reg.
		Normal	Cooperative	
57	Phenylalanine	−0.83 ± 0.25	−0.24 ± 0.22	1
105	Arginine	−0.19 ± 0.13	−2.41 ± 0.58	1
106	Arginine	−0.80 ± 0.33	−0.70 ± 0.20	1
108	Phenylalanine	−0.73 ± 0.24	0.06 ± 0.03	1
119	Serine	−0.42 ± 0.28	−0.78 ± 0.46	1
212	Arginine	−3.10 ± 0.66	−3.83 ± 1.00	2 and 3
215	Phenylalanine	−1.89 ± 0.39	−0.43 ± 0.22	2 and 3
301	Isoleucine	−0.64 ± 0.26	−0.46 ± 0.14	4
304	Phenylalanine	−1.56 ± 0.42	−1.07 ± 0.33	4
305	Alanine	−1.42 ± 0.17	−1.30 ± 0.26	4
309	Threonine	−0.77 ± 0.21	−0.37 ± 0.36	4
369	Isoleucine	−1.12 ± 0.43	−0.95 ± 0.28	5
370	Alanine	−0.73 ± 0.17	−1.19 ± 0.46	5
371	Methionine	−1.75 ± 0.29	−0.30 ± 0.07	5
372	Arginine	−4.49 ± 0.58	−1.86 ± 0.59	5
373	Leucine	−0.78 ± 0.17	−1.56 ± 0.41	5
374	Glutamic acid	2.48 ± 0.79	0.44 ± 0.56	5
482	Leucine	−1.24 ± 0.49	−2.26 ± 0.36	6

### 2.5. Pi-Pi Stacking in the Cooperative Binding Models

According to our previous studies [17,18], pi-pi stacking interactions are essential for multiple substrate binding. In human CYP2E1, Phe478 can form pi-pi stacking interactions with both substrates, acting as a structural linker for both substrates and decreasing the hydroxylation efficiency of the first substrate by positioning it in an unfavorable orientation in the active site. However, in the current study, we could not find such residue employing similar functions with Phe478 in human CYP2E1. Instead, a multiple pi-pi stacking mechanism was detected to be associated with the cooperative binding in CYP3A4.

**Figure 5:**
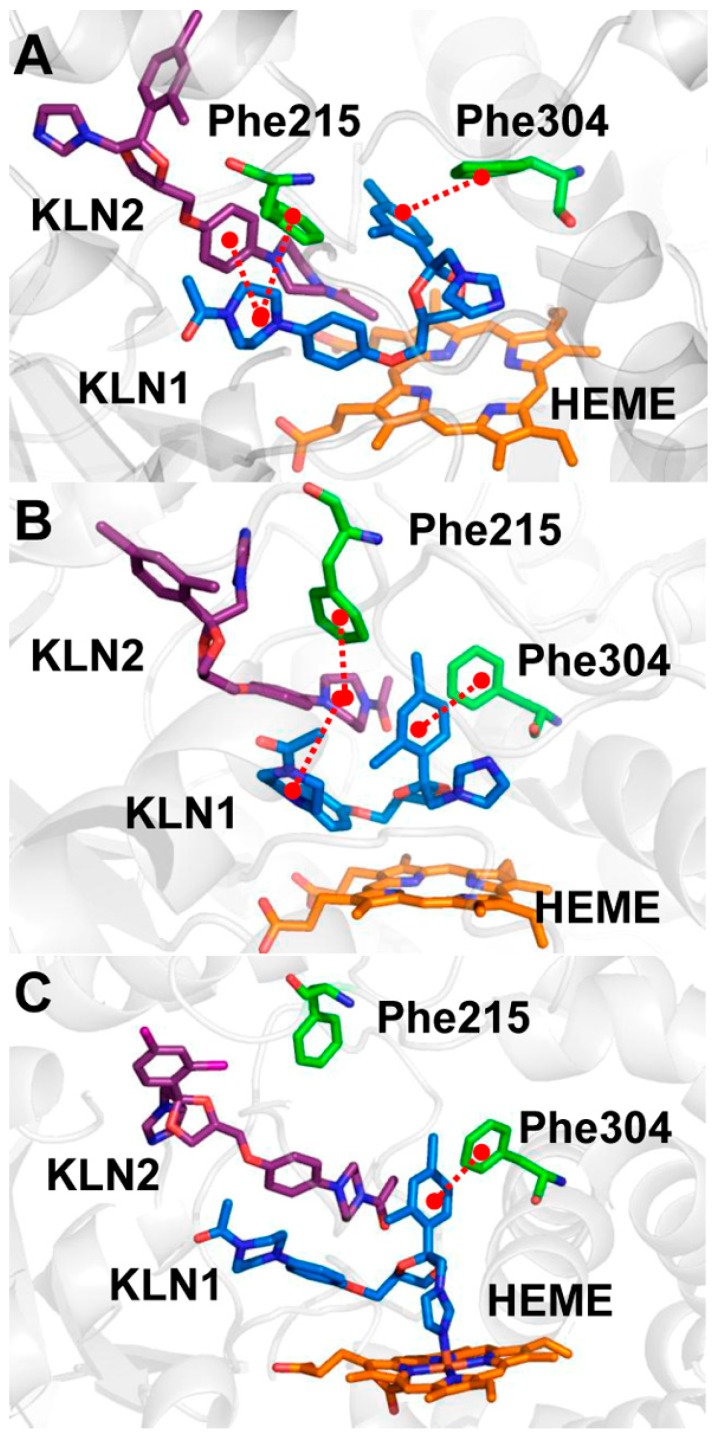
Pi-pi stacking interactions between Phe215, Phe304 and KLNs in the cooperative binding model of (**A**) the CYP3A4t structure; (**B**) the CYP3A4w structure; and (**C**) the crystal structure 2v0m.

According to the previous studies, if the distance between the aromatic rings of two different molecules is less than 7 Å, it is believed that they employed pi-pi stacking interactions [23,24]. In both CYP3A4t and CYP3A4w structure, two residues (Phe215 and Phe304) were found to have significant pi-pi stacking interactions with KLN in the normal model. The former functioned to stabilize KLN piperazine moiety, while the latter could recognize and position the phenyl group in KLN dioxolan moiety. When the second KLN bound into the active site, a 3-ring pi-pi stacking was found between Phe215 and the piperazine moieties of the two KLN molecules (Figure 5). The distances between the phenyl rings of the first KLN and Phe215 in the cooperative binding model (5.07 ± 0.32 Å in CYP3A4t structure and 4.86 ± 0.38 Å in CYP3A4w structure) was shorter than that in the normal binding model (5.53 ± 0.38 Å in CYP3A4t structure and 6.42 ± 0.36 Å in CYP3A4w structure, also see Figure S2), indicating that the first KLN moved toward Phe215 in the cooperative binding model. However, the dihedral angles between the phenyl rings of the first KLN and Phe215 were almost the same (~40°) in both normal and cooperative binding models (Figure S3). The shorter distance and identical dihedral angles indicated that the pi-pi stacking interaction formed by Phe215 became a little stronger in the cooperative binding models.

However, the pi-pi stacking between Phe304 and dioxolan moiety of the first KLN was enhanced in the cooperative binding model. The distance between phenyl rings in this pi-pi stacking in the cooperative binding model was 4.99 ± 0.27 Å in the CYP3A4t model and 5.05 ± 0.31 Å in the CYP3A4w model, much shorter than that in the normal binding model (5.39 ± 0.39 Å in CYP3A4t model and 5.11 ± 0.30 Å in CYP3A4w model). This pi-pi stacking interaction was essential for aromatic substrate, *i.e.*, aflatoxin B1 (AFB1). Computational studies showed that Phe304 could form a pi-pi stacking interaction with the first AFB1 to form a face-on C8-C9 epoxidation mode [25]. This binding mode was in good agreement with the available experiments and kinetic data, providing a theoretical insight into the mechanism of the region- and stereoselectivity of AFB1 oxidations. In addition, the dihedral angle between these two phenyl rings in the cooperative binding was ~31° in CYP3A4t structure and ~16° in CYP3A4w structure, much smaller than the normal binding model (~58° in CYP3A4t structure and ~52° in CYP3A4w structure, also see Figure S4).

This observation indicated that the phenyl rings in the dioxolan moiety of the first KLN adopted quite different conformations in the normal and cooperative binding models. In the normal binding model, this phenyl ring was perpendicular to the aromatic ring of Phe304. However, it became parallel to the phenyl group of Phe304 in the cooperative binding model. According to Tauer and co-workers [26], the parallel conformation employed more favorable free energies (~−5.88 kcal/mol) than the perpendicular conformation (~−4.71 kcal/mol). By looking into the simulation trajectories, we found that the parallel conformation of the dioxolan moiety of the first KLN were caused by the hydrophobic interactions of the second KLN (Figure 5).

## 3. Materials and Computational Methods

### 3.1. Constructing Computational Models

The initial structures of human CYP3A4 were abstracted from the crystal structures with PDB entries of 1tqn [27] and 1w0f [28]. Except for protein atoms and the heme group, all others (including crystal waters and metal ions) in these crystal structures were removed to yield apo models. The non-polar hydrogen atoms in apo models were also deleted. pKa values for each residue in apo models were calculated by Delphi [29] as a Poisson Boltzmann solver with a dielectric constant of 4. The hydrogen atoms were then added to the protein structure using t-leap module implemented in Amber 9.0 [30] based on the computational pKa values mentioned above. After energy minimization processes, the computational models were equilibrated by a short molecular dynamics simulation (~1 ns).

The antifungal drug ketoconazole (KLN) was selected as a typical inhibitor of CYP3A4 to build the normal and cooperative binding modes. Ten conformations were randomly selected from the molecular dynamics trajectories of the apo models in the equilibrium stage. The first KLN molecule was then docked into the active site of these conformations by AutoDock 4.0 [31]. The genetic algorithm was employed to generate 1000 independent results for each selected conformation. For each conformation, the one with KLN in the favorable orientation for oxidation and lowest binding energy was selected as normal binding models for further simulations. The second KLN molecule was then docked into the normal binding models to yield cooperative binding models, with a similar docking procedure of the normal binding models.

### 3.2. Molecular Dynamics Simulation

All the computational models were solvated in a specific simulation box with explicit TIP3P waters. The protein atoms and KLN molecule were parameterized by Amber force field parameters [32]. The force field parameters for heme group in CYP3A4 were obtained from previous theoretical study [33]. After solvation, all the models were subjected to steepest descent energy minimization (3000 steps), followed by conjugate gradient for the next 3000 steps and subsequently equilibrated with the protein backbone atoms fixed by a short molecular dynamics simulation (~1 ns) to reduce the van der Waals conflicts.

Finally, a 10-ns molecular dynamics simulation was performed by Amber 9.0 package with normal temperature, periodic boundary condition and NPT ensemble. During the molecular dynamics simulation, the pressure and temperature were set to 1 bar and 310 K, respectively. To maintain all the systems at a constant pressure and temperature, the Berendsen thermostat was applied with coupling times of 1.0 ps and 0.1 ps. The SHAKE algorithm was applied to constrain all bonds in all the models, and atoms velocities for start-up runs were obtained according to the Maxwell distribution at 310 K. PME algorithm was used to treat the electrostatic interactions, and the van der Waals interactions were calculated with a cutoff of 12 Å.

### 3.3. Binding Free Energy Estimation

The molecular mechanics Poisson Boltzmann surface area (MM-PB/SA) and molecular mechanics generalized Born surface area (MM-GB/SA) approaches implemented in Amber 9.0 was applied to estimate the binding free energy for the first KLN in the active site. The principles of the MM-PB/SA and MM-GB/SA methods can be summarized as:

(1) $$\text{Δ}G_{bind}=G_{complex}-\left(G_{protein}+G_{KLN}\right)\;$$

(2) $$\text{G}≅{\text{E}}_{gas}-\text{T}S_{config}+G_{sol}\;$$

(3) $$E_{gas}=E_{bond}+E_{ang}+E_{tor}+E_{vdw}+E_{ele}\;$$

(4) $$G_{sol}=G_{ele}+G_{non-polar}\;$$

In the current study, the binding free energy (ΔG_bind_) is treated as the difference value between the free energies of the binding mode (G_complex_), CYP3A4 (G_protein_) and KLN (G_KLN_). The energy terms mentioned above were calculated through Equation (2). As the computational models involved in the current study had similar entropy; the entropy contributions were neglected. Internal energy in the gas phase (E_gas_) is a standard force field energy calculated from Equation (3) by the strain energies from covalent bonds (E_bond_ and E_ang_), torsion angle (E_tor_), non-covalent van der Waals (E_vdw_) and electrostatic energy (E_ele_). The solvation free energy (G_sol_) is estimated based on an electrostatic term G_ele_ and a nonpolar component G_non-polar_ (Equation (4)). Either MM-PB/SA or MM-GB/SA approach can be used to compute the electrostatic term G_ele_. Molecular solvent accessible surface area (SASA) is employed to estimate the nonpolar component G_non-polar_. To analyze the residue contributions to the binding free energy, we also performed free energy decomposition based on the MM-PB/SA and MM-GB/SA calculations [34,35].

## 4. Conclusions

To study the cooperative binding mechanism of human CYP3A4, we constructed both normal and cooperative binding models for CYP3A4 with antifungal drug ketoconazole (KLN) based on the crystal structures 1tqn and 1w0f. Based on molecular dynamics simulation and free energy calculation of both normal and cooperative binding models, we found that the second KLN molecule had a significantly positive impact on the first one binding in the active site, so as to speed up its metabolism. This positive impact was mediated by two significant pi-pi interactions. The first pi-pi interaction (contributed by Phe215, the piperazine moiety of the first KLN, and the dioxolan moiety of the second KLN) functioned to position the first KLN in a favorable orientation for further metabolic reactions. The second pi-pi interaction (formed by Phe304 and the dioxolan moiety of the first KLN) could be enhanced by cooperative binding by making the phenyl group parallel to the aromatic ring of Phe304. The parallel conformation employed stronger binding free energy than the perpendicular conformation in the normal binding model.

## Supplementary Materials

Supplementary materials can be accessed at: http://www.mdpi.com/1420-3049/20/05/7558/s1.
