# Supplementary material for: Pi-pi Stacking Mediated Cooperative Mechanism for Human Cytochrome P450 3A4
Source: Molecules. 2015 Apr 24;20(5):7558–73. doi: 10.3390/molecules20057558 (PMC6272561; doi:10.3390/molecules20057558)
Supplement: Supplementary file 1 [file molecules-20-07558-s001.pdf]

Supplementary Materials

Table S1. The detailed information for the computational models involved in the current study. The crystal structures 1tqn.pdb and 1w0f.pdb were used to construct the normal (Model 1 and Model 3) and cooperative (Model 2 and Model 4) binding models.

Model	Protein Structure	Heme	1st KLN	2nd KLN	No. of Waters
1	1tqn.pdb	+	+	—	6421
2	1w0f.pdb	+	+	—	5976
3	1tqn.pdb	+	—	+	6403
4	1w0f.pdb	+	—	+	5987

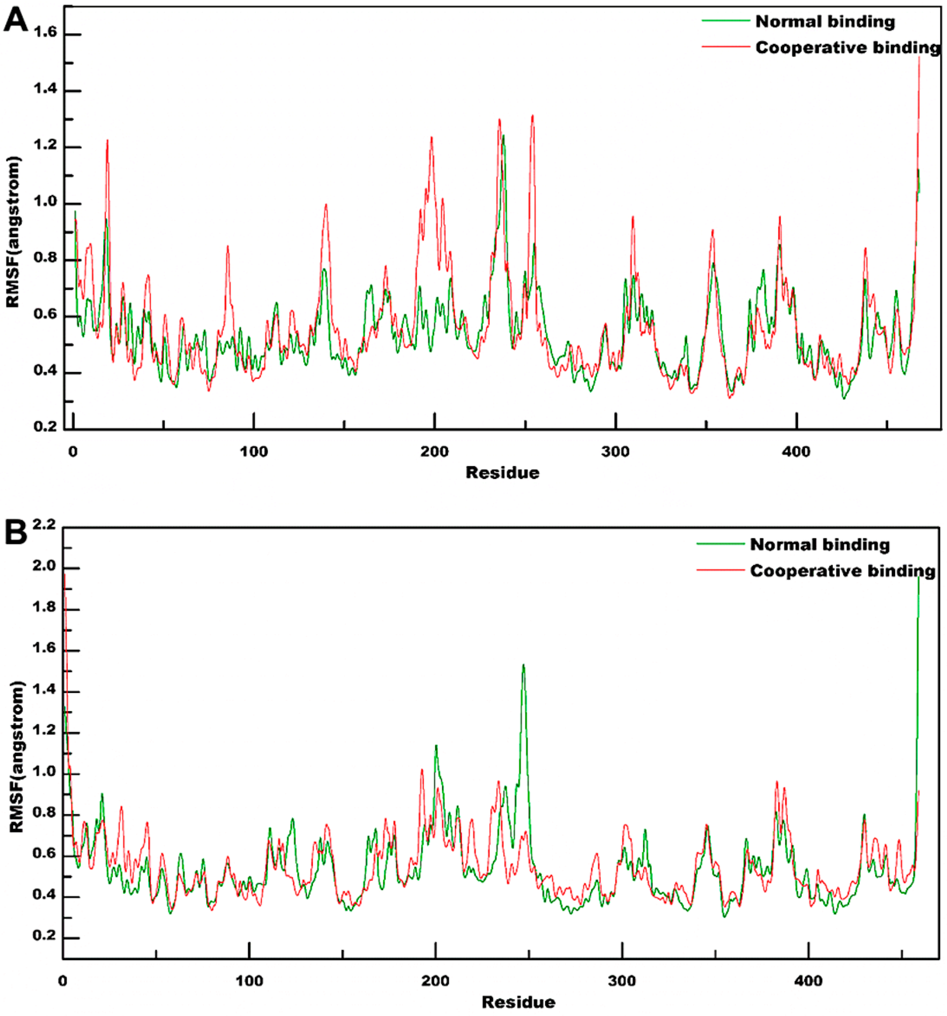

Figure S1. RMS fluctuations for protein backbone structure of (**A**) CYP3A4t structure and (**B**) CYP3A4w structure.

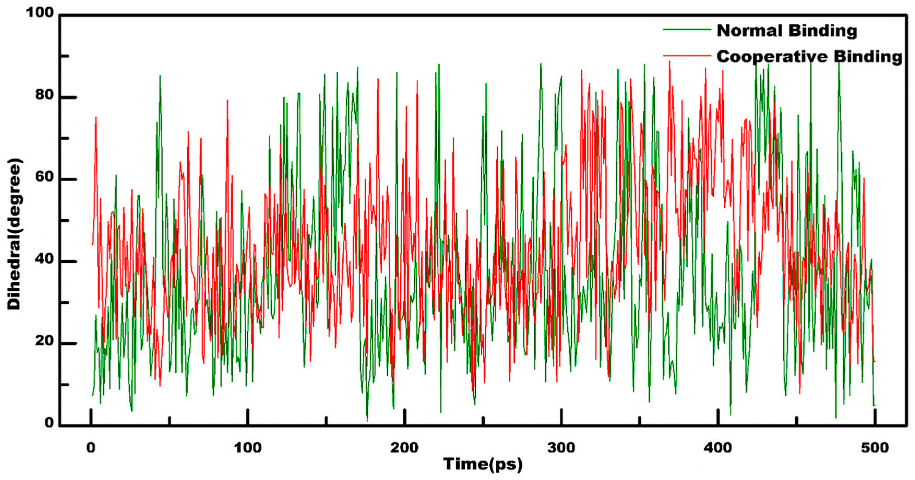

Figure S2. Dihedral angles between the phenyl rings of the first KLN and Phe215 for both normal and cooperative binding models in CYP3A4t structure.

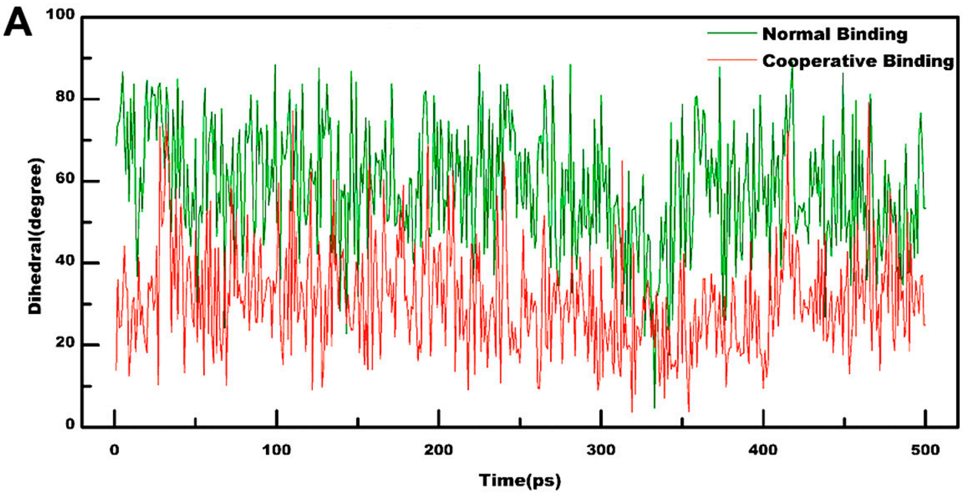

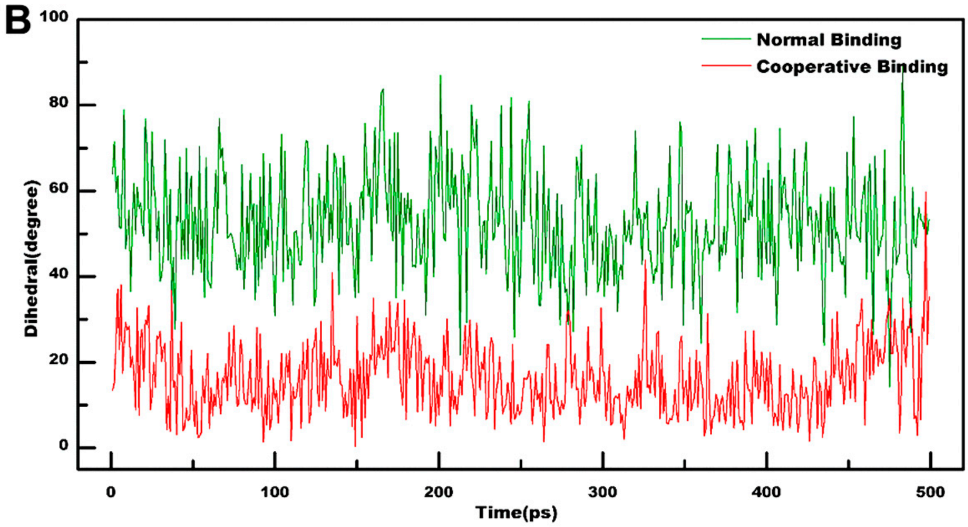

Figure S3. Dihedral angles between Phe304 and dioxolan moiety of the first KLN in (**A**) CYP3A4t structure and (**B**) CYP3A4w structure.
